# Electrocatalytic activity of lithium polysulfides adsorbed into porous TiO_2_ coated MWCNTs hybrid structure for lithium-sulfur batteries

**DOI:** 10.1038/srep40679

**Published:** 2017-01-18

**Authors:** Xiulin He, Huijie Hou, Xiqing Yuan, Long Huang, Jingping Hu, Bingchuan Liu, Jingyi Xu, Jia Xie, Jiakuan Yang, Sha Liang, Xu Wu

**Affiliations:** 1 School of Environmental Science and Engineering, Huazhong University of Science and Technology (HUST), Wuhan, 430074, P R China; 2 School of Electrical & Electronic Engineering, Huazhong University of Science and Technology (HUST), Wuhan, 430074, P R China

## Abstract

Lithium-sulfur batteries have attracted great attention because of their high energy density, environmental friendliness, natural abundance and intrinsically low cost of sulfur. However, their commercial applications are greatly hindered by rapid capacity decay due to poor conductivity of electrode, fast dissolution of the intermediate polysulfides into the electrolyte, and the volume expansion of sulfur. Herein, we report a novel composite MWCNTs@TiO_2_-S nanostructure by grafting TiO_2_ onto the surface of MWCNTs, followed by incorporating sulfur into the composite. The inner MWCNTs improved the mechanical strength and conductivity of the electrode and the outer TiO_2_ provided the adsorption sites to immobilize polysulfides due to bonding interaction between TiO_2_ and polysulfides. The MWCNTs@TiO_2_-S composite with a mass ratio of 50% (MWCNTs in MWCNTs@TiO_2_) exhibited the highest electrochemistry performance among all compositing ratios of MWCNTs/TiO_2_. The performance improvement might be attributed to the downward shift of the apparent Fermi level to a more positive potential and electron rich space region at the interface of MWCNTs-TiO_2_ that facilitates the reduction of lithium polysulfide at a higher potential. Such a novel hybrid structure can be applicable for electrode design in other energy storage applications.

In response to the emerging environmental and ecological concerns of using fossil fuels and the depleting of fossil fuel reserves, there is a growing demand on the development of renewable, environmental friendly and high energy density energy-storage technologies^1^,^2^,^3^. Rechargeable batteries, especially lithium-ion batteries, have become a key component in electrical vehicles and portable personal electronics due to their high specific energy density^1^,^2^,^3^,^4^,^5^. However, the practical applications of lithium-ion batteries are still greatly hindered by the limited theoretical capacities^3^,^6^,^7^,^8^. Lithium-sulfur batteries, with significant high theoretical capacity (1675 mAh g^−1^) and specific energy density (2600 Wh kg^−1^), are considered to be one of the most promising alternatives for next-generation energy storage devices. In addition, sulfur bears the advantages of low cost, non-toxic, and natural abundance, which make lithium-sulfur battery an attractive candidate for practical applications with mass production^9^,^10^,^11^,^12^.

Currently, several inherent issues of lithium-sulfur batteries should be solved before achieving satisfied performance: i. electric/ionic insulating of elemental sulfur and the discharge products (Li_2_S_2_ or Li_2_S)^13^, leading to poor electrical conductivity of electrode and low sulfur utilization; ii. polysulfides can be easily dissolved into the organic electrolyte and diffuse to the lithium anode, causing undesired parasitic reaction; iii. the shuttle effect leading to random precipitation of Li_2_S_2_ or Li_2_S on the lithium anode, which dramatically changes the electrode morphology and accelerates capacity fading^14^; iv. large volumetric expansion (about 80%) upon the charge/discharge process, resulting in the destruction of the electrode, and finally the capacity decay of the battery^15^.

To address these problems, several strategies have been proposed to enhance the performance of lithium-sulfur batteries. Some metal oxides with polar surfaces, including Mg_0.6_Ni_0.4_O, Al_2_O_3_, MnO_2_ and TiO_2_^16^,^17^,^18^,^19^ have been used as additives or absorbents to confine sulfur and polysulfides. Porous TiO_2_, for example, can serve as on-site adsorption host for encapsulating polysulfides through bonding interaction to minimize the dissolution of sulfur into the electrolyte^20^. Yi Cui and co-workers also reported that conductive Ti_4_O_7_ was a highly effective matrix to bind with sulfur species due to the strong adsorption of polysulfides on the low-coordinated Ti sites of Ti_4_O_7_^21^. However the relatively low conductivity of metal oxides makes it insufficient to satisfy the high rate performance for lithium-sulfur batteries. In fact, various carbon matrix, including mesoporous carbon or microporous carbon^22^,^23^,^24^,^25^, graphene oxide^26^,^27^,^28^, carbon nanotube or carbon nanofiber^29^,^30^,^31^, and hollow carbon sphere^32^,^33^ have also been used as conductive matrix in lithium-sulfur batteries due to the conductivity of carbon and the physical confinement property of the nanostructured carbon. Therefore the combination of the conductive network, such as MWCNTs^34^, with metal oxides would be an ideal route to overcome the low conductivity issue associated with metal oxides. Other strategies such as optimization of organic electrolyte^35^, sulfur loading conducting polymer^36^, designing new Li–S batteries device configuration^37^ were also employed and stable cycle performance was achieved.

Herein, we report a novel MWCNTs@TiO_2_-S hybrid nanostructure by grafting TiO_2_ onto the exterior surface of MWCNTs, followed by incorporating sulfur through the melt-diffusion method. TiO_2_ provided adsorption sites to bind with polysulfides, while MWCNTs acted as the efficient current collector. The hybrid structure of TiO_2_ coated MWCNT demonstrated the synergistic effect towards electron transfer kinetics for the redox response of polysulfides and an interpretation based on bending of energy bands at the MWCNT-TiO_2_ interface was also proposed.

## Results and Discussion

### The structural characterization

**Figure 1:**
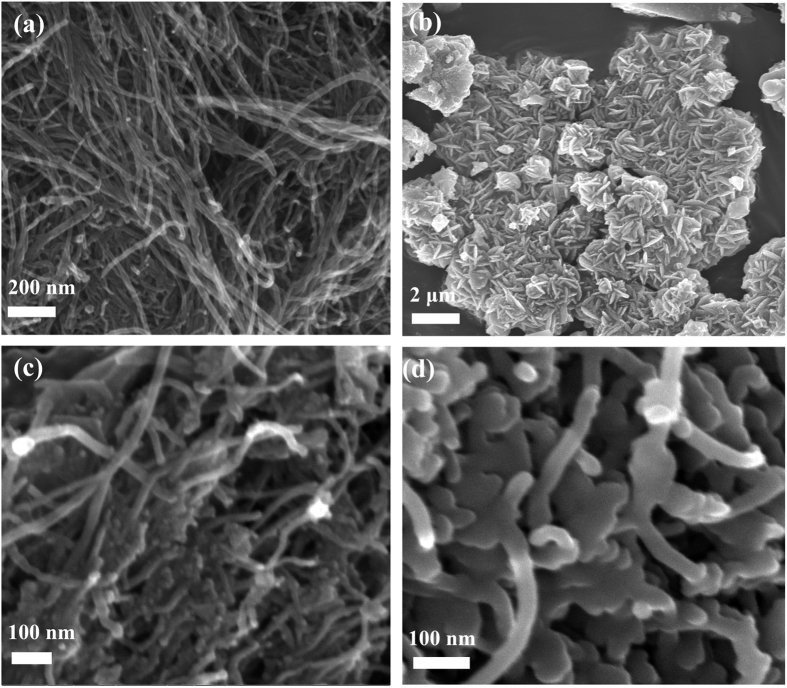
SEM images of (**a**) MWCNTs, (**b**) TiO_2_, (**c**) 50%-MWCNTs@TiO_2_ and (**d**) 50%-MWCNTs@TiO_2_-S.

Scanning electron microscopy (SEM) was used to characterize the morphologies of the pristine MWCNTs, TiO_2_, 50%-MWCNTs@TiO_2_ and 50%-MWCNTs@TiO_2_-S composites (50% refers to the mass proportion of MWCNTs in the MWCNTs@TiO_2_ composite). MWCNTs exhibit smooth surface and one dimensional linear structure, with an outer diameter of about 20 nm and a length of about several microns (Fig. 1a). TiO_2_ reveals grain-like morphology with the average length of about 300 nm (Fig. 1b), the fine flower-like nanostructure indicates high specific surface area^38^. The SEM image of 50%-MWCNTs@TiO_2_ shows TiO_2_ nanoparticles coating on the surface of MWCNTs with a rougher surface (Fig. 1c) comparing with pristine MWCNTs. Interestingly, in contrast to the aggregation of the flower-like morphology of TiO_2_ particles in the absence of MWCNTs, TiO_2_ is distributed uniformly on the exterior of MWCNTs, probably due to the enhanced nucleation of nanoparticles on hydrophilic surface resulting from carboxylation of MWCNTs^39^. After encapsulating sulfur into the 50%-MWCNTs@TiO_2_, the surface of 50%-MWCNTs@TiO_2_-S becomes smoother without agglomeration (Fig. 1d), indicating the successful incorporation of sulfur into the composites.

**Figure 2:**
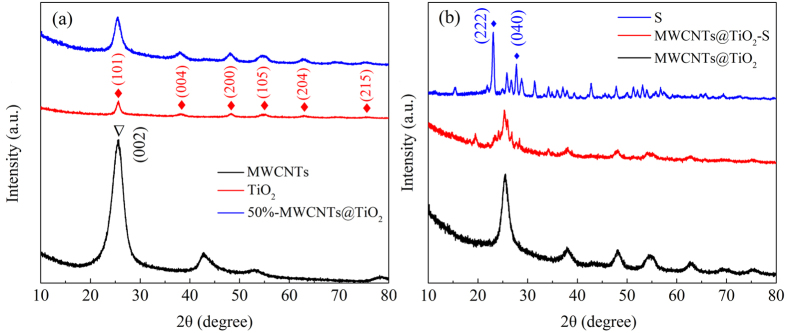
(**a**) XRD patterns of MWCNTs, TiO_2_ and 50%-MWCNTs@TiO_2_; (**b**) XRD patterns of pure sulfur, 50%-MWCNTs@TiO_2_ and 50%-MWCNTs@TiO_2_-S.

To further confirm the nanostructure of the composites, X-ray diffraction (XRD) patterns were acquired for MWCNTs, TiO_2_, 50%-MWCNTs@TiO_2_ and 50%-MWCNTs@TiO_2_-S respectively (Fig. 2). It reveals an apparent high-intensity graphitic peak at 2θ value of around 26°, which can be ascribed to the (002) reflection of MWCNTs^34^, and 2θ values of 25.3°, 37.8°, 48.0°, 53.9°, 62.7°, 75.0° are attributed to the (101), (004), (200), (105), (204) and (215) crystalline phases of anatase TiO_2_ (JCPDF NO. 21-1272)^40^ (Fig. 2a). After coating MWCNTs with TiO_2_, MWCNTs peaks become much weaker, while the peaks of anatase TiO_2_ remain strong. From the XRD patterns of pure sulfur, 50%-MWCNTs@TiO_2_ and 50%-MWCNTs@TiO_2_-S (Fig. 2b), intense and sharp diffraction peaks of sulfur at 2θ values of 23.4° and 28.0° were observed, which match well with the (222) and (040) reflections of the Fddd orthorhombic phase (JCPSD no. 08-0247)^41^, indicating a well-defined crystal structure. After sulfur being encapsulated into the 50%-MWCNTs@TiO_2_, almost all typical sulfur diffraction peaks and the anatase TiO_2_ peaks became weaker, indicating that sulfur was dispersed homogeneously into the composite and no obvious aggregates of sulfur or TiO_2_ on the surface of MWCNTs, which is consistent with the results from SEM.

Thermal gravimetric analysis (TGA) was utilized to evaluate the content of sulfur incorporated into the composites (Figure S1). All materials show a plateau of weight loss from 200 °C to 350 °C, reflecting the evaporation of sulfur loaded in the composites. The sulfur contents were determined to be 65.95%, 58.78%, 65.62%, 61.66% and 62.99% respectively, which are lower than the proportion of sulfur and MWCNTs@TiO_2_ (75%) utilized for incorporating sulfur into the MWCNTs@TiO_2_, indicating that a small amount of excessive sulfur was removed through the heat treatment process and no excess sulfur remained on the surface.

### Electrochemical performance of the MWCNTs@TiO_2_-S composites

**Figure 3:**
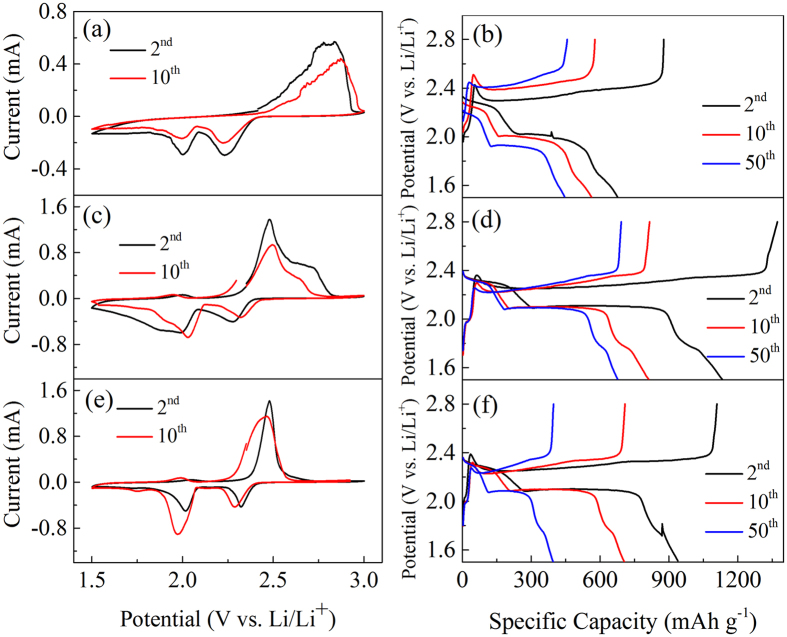
Comparison of CV curves and voltages vs. capacity profiles at 0.1 C of (**a,b**) 0%-MWCNTs@TiO_2_-S, (**c,d**) 50%-MWCNTs@TiO_2_-S and (**e,f**) 100%-MWCNTs@TiO_2_-S in the voltage range of 3.0–1.5 V vs Li/Li^+^.

The electrochemical features of different composites can be seen from the cyclic voltammetry curves (CV) recorded at a scan rate of 0.1 mV s^−1^ in the voltage range of 3.0–1.5 V. As the first cycle showed the activation behavior, the second and the tenth CV curves were selected to evaluate the redox behaviors of the cathodes (Fig. 3a,c,e). During the discharging process, all electrodes show two apparent reduction peaks at the same potential for the second and tenth cycle, indicating excellent cycling stability. The peak at around 2.3 V represents the reduction of element sulfur (S_8_) to high-order lithium polysulfide (Li_2_S_n_, 4 ≤ n ≤ 8), while the peak at around 2.0 V corresponds to the reduction of high-order lithium polysulfide to low-order lithium polysulfide (Li_2_S_2_ and Li_2_S). In the subsequent anodic process, an oxidation peak develops at around 2.5 V of 0%-MWCNTs@TiO_2_-S and 100%-MWCNTs@TiO_2_-S (Fig. 3a,e), which shows similar oxidation potential for typical microporous and mesoporous carbon structures^24^,^42^. Interestingly, 50%-MWCNTs@TiO_2_-S shows an additional oxidation peak at about 2.7 V (Fig. 3c), which have only been observed in a few sulfur-conductive matrix systems with high conductivity and low polarization^26^,^32^, and this oxidation peak is associated with the electrocatalytic oxidation towards the Li-S redox reaction due to the synergistic effect of the best combination of TiO_2_ and MWCNTs (with the mass ratio of 50%), which further promoted the electron transfer rate and minimized the polarization of the battery^21^,^43^,^44^. The charge - discharge curves of different electrodes from the 2^nd^, 20^th^ and 50^th^ cycles demonstrate two distinct discharge plateaus for all tested cathodes (Fig. 3b,d,f), including a shorter and higher potential plateau at 2.3 V and a prolonged lower potential plateau at around 2.0 V, in agreement with their redox peaks of CV curves respectively. Other composites (20%-MWCNTs@TiO_2_-S, 70%-MWCNTs@TiO_2_-S) were also studied (Figure S2), both of which have the same two reduction peaks at around 2.3 V and 2.0 V, one oxidation peak at 2.5 V, and the optimal performance was observed with the mass ratio of 50% for MWCNTs.

**Figure 4:**
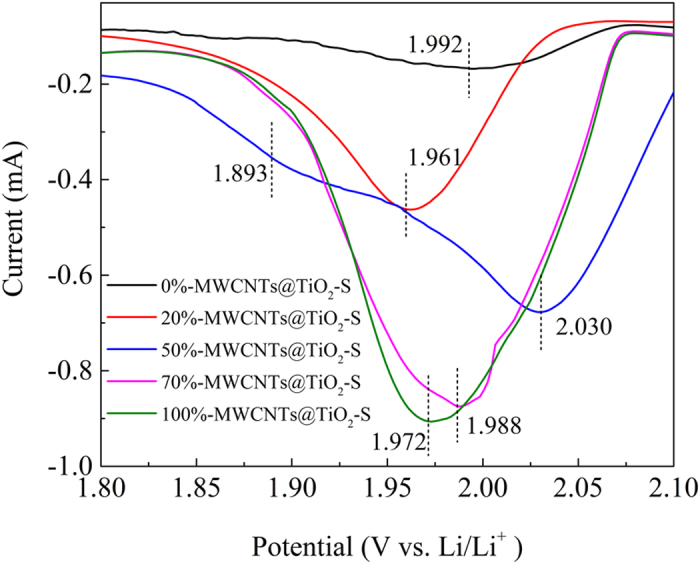
The partial enlarged drawing of the reduction peaks of varied MWCNTs loading ratios (0%, 20%, 50%, 70%, and 100%).

50%-MWCNTs@TiO_2_-S composite shows a reduction peak at higher reduction potential (2.03 V) comparing to electrodes with other TiO_2_ loading ratios (Fig. 4), which indicates improved electrochemical kinetics and better utilization of active mass^21^,^45^. The improvement can be attributed to the immobilization of polysulfides on the TiO_2_ surface, as well as the nature of energy band bending on the interface of MWCNTs and TiO_2_. It have been reported that TiO_2_ are terminated with hydrophilic Ti-O groups and surface hydroxyl groups, which are known to bind favorably with polysulfides anions^46^. In fact, Li_2_S_x_ shows strong adsorption on TiO_2_ (101) from DFT calculations, especially for Li_2_S_2_ and Li_2_S_4_, and the chemical bonding between Ti-S bonds and S-O bonds were detected from XPS analysis, confirming that sulfur atom in polysulfides is bonded strongly with two bridging oxygen atoms on the TiO_2_ surface^21^. Hence the soluble high order lithium polysulfides (Li_2_S_n_, 4 ≤ n ≤ 8) can be anchored at the adsorption site, significantly alleviating the undesired shuttle effect^47^,^48^. In addition, Li_2_S_x_ shows molecular interaction with oxygen vacancies in TiO_2_^49^,^50^. To characterize the density of oxygen vacancies, electron paramagnetic resonance (EPR) spectroscopy was carried out to evaluate the unpaired electrons associated with oxygen vacancies in the TiO_2_ crystalline structure (Figure S3). The strong peak at g = 2.001 was attributed to oxygen vacancies in the TiO_2_ crystalline structure and the density of oxygen vacancy was estimated to be 1.09 × 10^11^ cm^−3^, which provides additional adsorption sites for polysulfides^49^.

**Figure 5:**
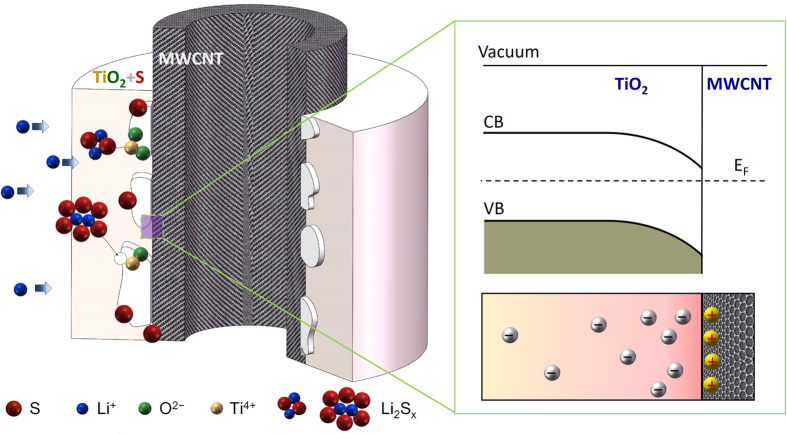
Schematic diagram for the adsorption mechanism of lithium polysulfides on the TiO_2_, as well as energy levels and densities of free charge carriers close to the interface of TiO_2_-MWCNTs.

The composite 50%-MWCNTs@TiO_2_-S shows two reduction peaks at around 2.03 V and 1.89 V respectively, indicating the co-existence of two types of electron transfer kinetics. Anatase TiO_2_ is known to have a band gap of 3.2 V, with a Fermi level of 1.3 V (work function of 6.0 V vs. vacuum level of −4.7 V vs. NHE)^51^, which is more positive than the Fermi level of the metallic MWCNTs (0.2 V vs. NHE)^52^. When TiO_2_ is in contact with MWCNTs, electrons flow from MWCNTs to TiO_2_ until an equilibrium of Fermi energy is reached and the energy bands bend downwards at the interface, leaving an electron rich accumulation layer on the TiO_2_ side, and a hole rich surface at the MWCNTs side of the TiO_2_-MWCNTs interface (Fig. 5), which is consistent with the literature^53^. The downward shift of the apparent Fermi level to a more positive potential facilitates the reduction of lithium polysulfide at 2.03 V. To balance the negative charge at the TiO_2_ side, holes are accumulated on the MWCNTs surface at the interface (Fig. 5), shifting the reduction potential of lithium polysulfide to a more negative one at 1.89 V. The typical width of the space charge region is around tens of nanometers^54^,^55^. When it is in the same order with the thickness of the TiO_2_ coating, as the case of 50%-MWCNTs@TiO_2_-S, the electron rich space charge region may even expand from the interface to the exterior surface of the TiO_2_ layer, leading to a pronounced reduction peak at a relative positive potential (2.03 V). Further increasing of the TiO_2_ thickness may reduce the electron density on the exterior surface of the TiO_2_ coating (20%-MWCNTs@TiO_2_-S), and deteriorates the reduction kinetics. In summary, the results demonstrate that TiO_2_ and MWCNTs composite with a mass ratio of 50% (MWCNTs) may produce a synergistic effect that have a positive catalytic activity towards the redox property of polysulfides.

**Figure 6:**
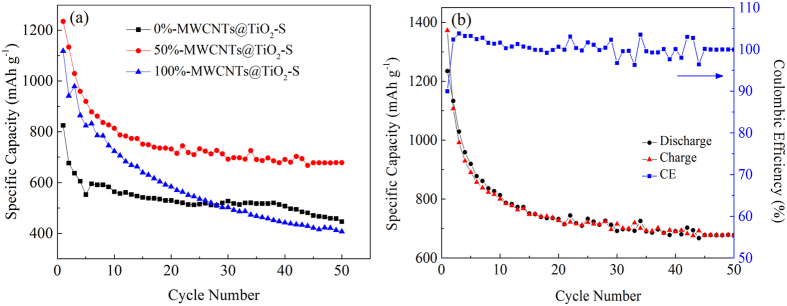
(**a**) Cycle performances at 0.1 C of MWCNTs@TiO_2_-S composites with different mass ratio of MWCNTs; (**b**) Cycle performance of 50%- MWCNTs@TiO_2_-S at 0.1 C.

Electrochemical performances of cathodes with various MWCNTs loading ratio (0%, 20%, 50%, 70%, and 100%) have been investigated by galvanostatic charge/discharge measurements. In this study, all specific capacity values were calculated on the basis of the mass of sulfur. After the first cycle of activation and stabilization, the discharge capacities achieve 677, 1133, and 941 mAh g^−1^ for 0%-MWCNTs@TiO_2_-S, 50%-MWCNTs@TiO_2_-S, 100%-MWCNTs@TiO_2_-S respectively (Fig. 6a). After 50 cycles, the capacities decrease to 446, 679, 408 mAh g^−1^, indicating that a higher performance retention for 50%-MWCNTs@TiO_2_-S after cycling. It is believed that the dissolution of polysulfides is the main challenge for capacity degradation. Capacity retention decreases with the decline of mass fraction of TiO_2_, which indicated that higher TiO_2_ mass ratio may lead to higher absorption ability toward sulfur and polysulfides^21^,^56^. However, higher mass fraction of TiO_2_ may also deteriorate the electrical conductibility of the cathode^57^. The results show that 50%-MWCNTs@TiO_2_-S might be the relatively optimal mass proportion, leading to the highest reversible capacity and excellent capacity retention. The coulombic efficiency (CE) of 50%-MWCNTs@TiO_2_-S remained above 95% over 50 cycles (Fig. 6b), indicating minimum shuttle effect of polysulfides and stable cycle performance. Other composites (20%-MWCNTs@TiO_2_-S, 70%-MWCNTs@TiO_2_-S) were also studied (Figure S4), and the results demonstrate that the mass ratio of 50% is still optimal.

**Figure 7:**
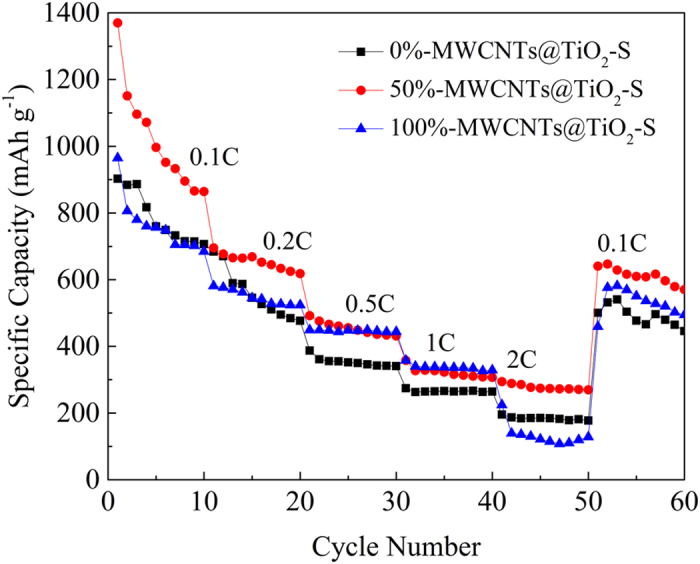
Rate properties of 0%-MWCNTs@TiO_2_-S, 50%-MWCNTs@TiO_2_-S and 100%-MWCNTs@TiO_2_-S composites at different current densities from 0.1 C to 2 C.

It is clear that the rate capability of 50%-MWCNTs@TiO_2_-S was much better than the 0%-MWCNTs@TiO_2_-S and 100%-MWCNTs@TiO_2_-S (Fig. 7). It demonstrates a discharge capacity of about 900 mAh g^−1^ at 0.1 C rate after 10 cycles. Subsequently, cyclings at 0.2 C, 0.5 C, 1 C and 2 C show high reversible capacities of 700 mAh g^−1^, 500 mAh g^−1^, 360 mAh g^−1^, 300 mAh g^−1^ respectively. And when the current rate switches from 2 C to 0.1 C again, the 50%-MWCNTs@TiO_2_-S reaches a capacity of 640 mAh g^−1^, revealing better stability of the cathode material. The higher electrochemical performance might be attributed to the synergetic effect of TiO_2_ and MWCNTs, which is consistent with the CV results.

**Figure 8:**
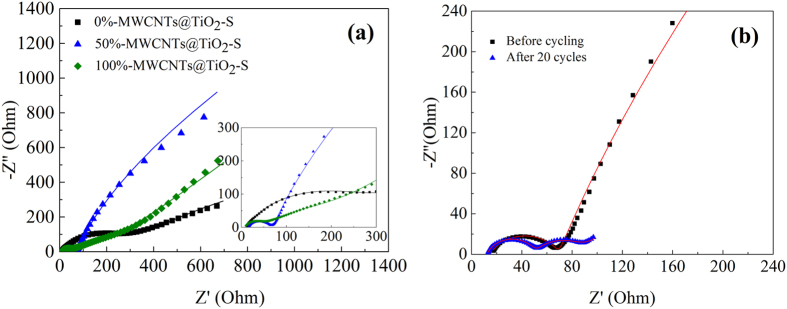
(**a**) EIS spectra of 0%-MWCNTs@TiO_2_-S, 50%-MWCNTs@TiO_2_-S, 100%-MWCNTs@TiO_2_-S composites electrodes before cycling, and (**b**) EIS spectra of 50%-MWCNTs@TiO_2_-S before and after the 20^th^ cycle. The experimental results were labeled with scatter plot and the fitting results were labeled with line.

In order to shed light on the role of TiO_2_ and MWCNTs in the electrochemical reaction, electrochemical impedance spectroscopy (EIS) spectra were acquired (Fig. 8) and analyzed by fitting with equivalent circuits (Figure S5). It can be seen that all EIS spectra were composed of a depressed semicircle in the high-frequency region before cycling, corresponding to the charge-transfer process generated at the electrode/electrolyte interface, and a sloping straight line in the low-frequency region corresponding to the Warburg diffusion process (Fig. 8a). The charge-transfer resistance (R_ct_) decreases with the increase of the mass ratio of MWCNTs, indicating that higher proportion of MWCNTs contributes to higher conductivity of the electrode. Moreover, after 20 cycles, the obtained impedance spectrum of 50%-MWCNTs@TiO_2_-S contains two semicircles (Fig. 8b), the one in the high-frequency reflects the charge-transfer resistance (R_ct_), while the other in the middle-frequency may be attributed to the formation of insoluble polysulfides species such as Li_2_S_2_/Li_2_S^58^. Previous report showed that the impedance of interfacial charge-transfer dominated the reduction reaction during the upper voltage plateau, whereas the mass transport dominated the lower voltage plateau^58^,^59^. After 20 cycles, the semicircle resistance in the high-frequency decreases (from 69 Ω to 46 Ω), indicating faster interfacial charge transfer owing to the synergistic effect of MWCNTs and TiO_2_.

## Conclusions

A nanostructure MWCNTs@TiO_2_-S composite with TiO_2_ nanoparticles coating on the surface of MWCNTs and sulfur homogeneously distributing into the porous TiO_2_ was designed as a novel cathode material for Li-S battery. The MWCNTs@TiO_2_-S composite with 50% MWCNTs exhibited higher electrochemistry performance comparing with other combination ratio of MWCNTs and TiO_2_ in the composites including 0%-MWCNTs@TiO_2_-S, 20%-MWCNTs@TiO_2_-S, 70%-MWCNTs@TiO_2_-S and 100%-MWCNTs@TiO_2_-S, with an initial discharge capacity of 1133 mAh g^−1^, and maintain 674 mAh g^−1^ after 50 cycles at 0.1 C. We ascribe the improvement of the electrochemistry performance of this composite to the unique hybrid nanostructure of MWCNTs@TiO_2_-S, and the synergistic effect of MWCNTs and TiO_2_. The inner MWCNTs can improve the mechanical strength and conductivity of the whole electrode, further ensure the fast electronic transport, while the outer porous TiO_2_ wrapping the exterior surface of MWCNTs can provide the adsorption sites to immobilize polysulfides. The improvement of the electrochemical performance might be attributed to the downward bending of energy band on the interface of MWCNTs and TiO_2_, and a charge region of rich electron developed at the TiO_2_ side of the interface that could even extend to the exterior TiO_2_ surface. The strategy employed in this work provides an efficient way of recapturing polysulfides from the electrolyte while maintaining the high conductivity and redox activity, which can also be applicable for designing electrode materials for other energy stordage applications.

## Methods

### Chemicals and Materials

Multi-walled carbon nanotubes (MWCNTs purity >95%) were purchased from Nanotech Port Co., Ltd. (Shenzhen, China). Nitric acid (HNO_3_ 65%) and dehydrated alcohol were supplied by Aldrich Chemical Company (America). Tetrabutyl titanate (TBT) was purchased from the Aladdin Industrial Corporation (America). All chemical reagents were analytical grade and were used as received.

### Materials synthesis

Prior to TiO_2_ coating, the pristine MWCNTs were pretreated in 6 M concentrated nitric acid and refluxed at 120 °C for 12 h to remove impurities and introduce carboxyl groups on the surface, subsequently lead to improved dispersion of MWCNTs. Then the acid-treated MWCNTs (with varied mass ratio) were dispersed in 50 mL ethanol under sonication for 30 min, after that, 1 mL tetrabutyl titanate (TBT) and 10 mL glycerol were added into the solution, and stirred for 30 min. Then the mixture was transferred into a 100 mL Teflon-lined stainless steel autoclave at 180 °C for 15 h. Subsequently, the precipitation was washed with ethanol and deionized water, dried at 60 °C overnight and calcined at 450 °C in ambient atmosphere for 2 h.

The MWCNTs@TiO_2_-S composites were prepared by a simple melt-diffusion method. Firstly, sulfur and MWCNTs@TiO_2_ were mixed by physical grinding at the weight ratio of 3:1, then the mixture was sealed in a Teflon-lined autoclave in an oven and heated at 155 °C for 12 h; followed by calcination at 250 °C in nitrogen atmosphere for 2 h to remove exterior excess sulfur. MWCNTs@TiO_2_-S were obtained after cooling down to room temperature.

### Preparation of the cathodes

All the cathodes were prepared by mixing the MWCNTs@TiO_2_-S, Super-P and PVDF (polyvinylidene fluoride) binder (80:10:10 by weight) in *N*-methyl-*2*-pyrrolidone solvent to form a slurry. The slurry was uniformly spread onto the Al foil and dried at 50 °C for 12 h in a vacuum oven to act as the working electrode. The mass loading of the active S was 2 mg cm^−2^. The 2032-type coin cells were assembled in an argon-filled glove box using Li metal foil as the counter electrode and the reference electrode. The electrolyte was 1.0 M lithium bis- (trifluoromethanesulfonyl)imide (LiTFSI) and 0.1 M LiNO_3_ in 1,3-dioxolane(1,3-DOL) and 1,2-dimethoxyethane(1,2-DME) (volume ratio, 1:1).

### Electrochemical measurements

Cyclic voltammetry (CV) measurements were performed with an electrochemical workstation (Biologic VSP 300) using a voltage range of 3.0−1.5 V vs. Li/Li^+^ at a scan rate of 0.1 mV s^−1^. Galvanostatic charge–discharge of the Li-S battery was conducted using Land battery system (CT2001A) operated at different rates between cut-off potentials of 3.0−1.5 V. Electrochemical impedance spectroscopy (EIS) was measured with the frequency range from 100 kHz to 10 mHz, and the voltage amplitude applied to the coin cells was 5 mV. Specific capacity values were calculated based on the mass of sulfur in the samples.

### Structural characterization

The morphology and microstructure were characterized using SEM operating at 10.00 kV (Sirion 200); the crystallographic structure was characterized using XRD (PANalytical B.V. X’pert PRO) operating at 40 kV and 40 mA using Cu Kα radiation (λ = 0.15406 nm); and the mass fraction of sulfur were estimated using TGA (Waters TGA Q 600), performed under an Ar atmosphere with a heating rate of 10 °C min^−1^ from room temperature to 600 °C.

## Additional Information

**How to cite this article**: He, X. *et al*. Electrocatalytic activity of lithium polysulfides adsorbed into porous TiO_2_ coated MWCNTs hybrid structure for lithium-sulfur batteries. *Sci. Rep.* **7**, 40679; doi: 10.1038/srep40679 (2017).

**Publisher's note:** Springer Nature remains neutral with regard to jurisdictional claims in published maps and institutional affiliations.

## Supplementary Material

Supplementary Information
