# Supplementary material for: Electrocatalytic activity of lithium polysulfides adsorbed into porous TiO2 coated MWCNTs hybrid structure for lithium-sulfur batteries
Source: Sci Rep. 2017 Jan 18;7:40679. doi: 10.1038/srep40679 (PMC5241630; doi:10.1038/srep40679)
Supplement: Supplementary Information [file srep40679-s1.pdf]

Supporting information

Electrocatalytic activity of lithium polysulfides adsorbed into porous TiO_2 coated MWCNTs hybrid structure for lithium-sulfur batteries

Xiulin He[1,#], Huijie Hou[1,#], Xiqing Yuan[1], Long Huang[1], Jingping Hu[1,*], Bingchuan Liu[1], Jingyi Xu[1], Jia Xie[2], Jiakuan Yang[1,*], Sha Liang[1], Xu Wu[1]

[1] *School of Environmental Science and Engineering, Huazhong University of Science and Technology (HUST), Wuhan, 430074, P R China*

[2] *School of Electrical & Electronic Engineering, Huazhong University of Science and Technology (HUST), Wuhan, 430074, P R China*

* Corresponding authors: Prof. Jingping Hu, E-mail: hujp@ hust.edu.cn, Prof. Jiakuan Yang, E-mail: jkyang@ hust.edu.cn, Tel: +86-27-87793948; Fax: +86-27-87792101

# These two authors contribute equally to this paper.

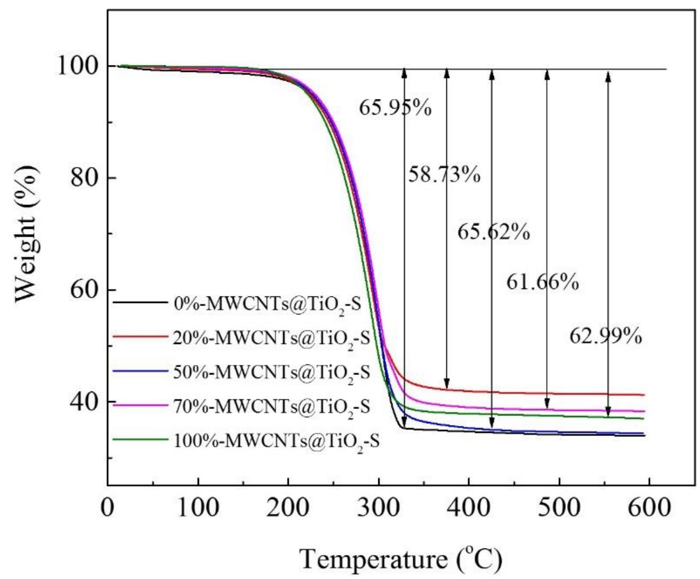

Figure S1. TGA curves of MWCNTs@TiO_2-S with varied mass ratios of MWCNTs (0%, 20%, 50%, 70% and 100% respectively).

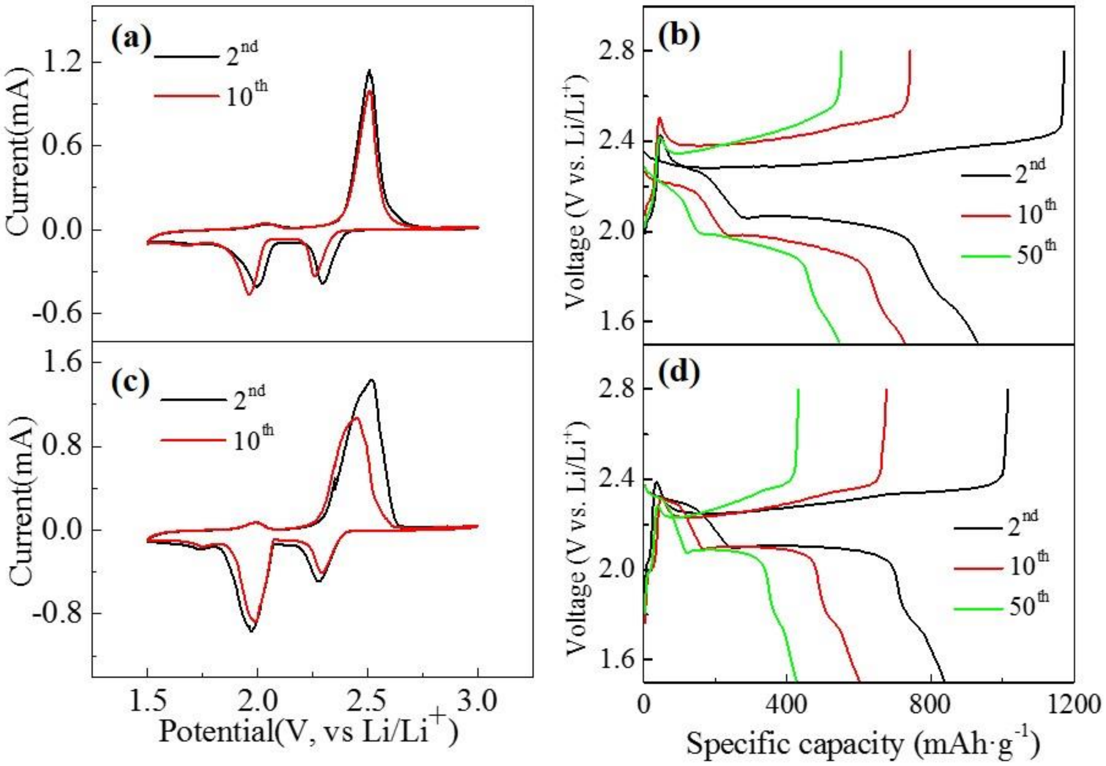

Figure S2. Comparison of CV curves and voltage vs. capacity profiles at 0.1 C of (a, b) 20%-MWCNTs@TiO_2-S, (c, d) 70%-MWCNTs@TiO_2-S in the voltage range of 3.0-1.5 V vs Li/Li^+.

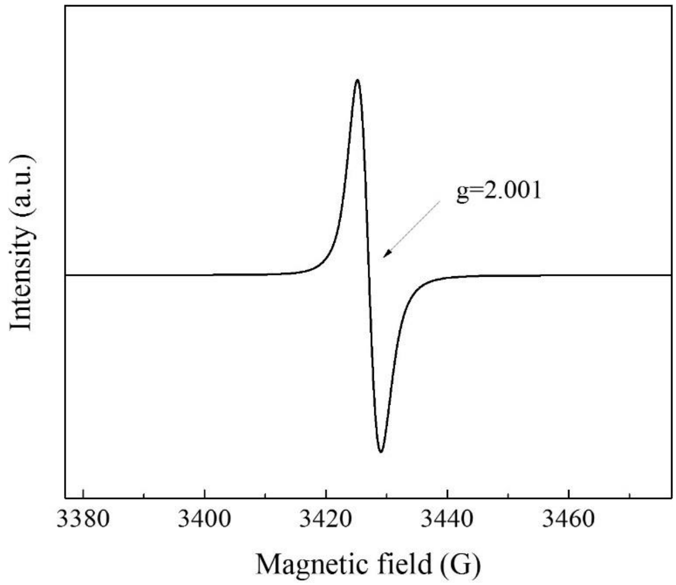

Figure S3. EPR spectroscopy of TiO_2 prepared using identical method to prepare MWCNTs@TiO_2.

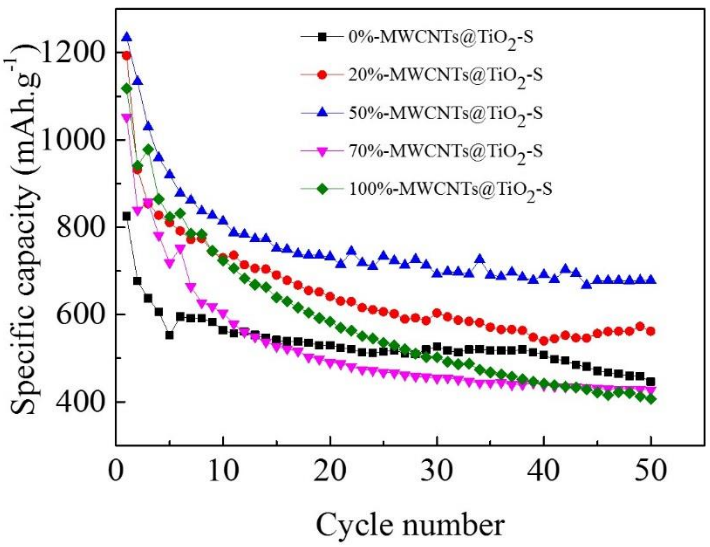

Figure S4. Cycle performances at 0.1 C of MWCNTs@TiO_2-S composites with different mass ratios of MWCNTs.

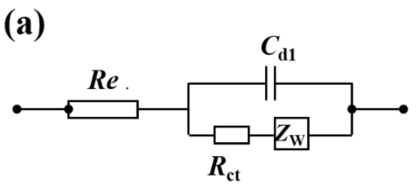

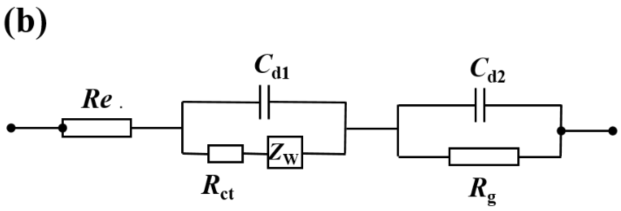

Figure S5. (a) The equivalent circuits used to fit the impedance spectra of Figure 8a and (b) The equivalent circuits used to fit the impedance spectra of Figure 8b.
